# Resurrection plants of the genus *Ramonda*: prospective survival strategies – unlock further capacity of adaptation, or embark on the path of evolution?

**DOI:** 10.3389/fpls.2013.00550

**Published:** 2014-01-10

**Authors:** Tamara Rakić, Maja Lazarević, Živko S. Jovanović, Svetlana Radović, Sonja Siljak-Yakovlev, Branka Stevanović, Vladimir Stevanović

**Affiliations:** ^1^Department of Plant Ecology and Phytogeography, Faculty of Biology, University of BelgradeBelgrade, Serbia; ^2^Laboratory for Plant Molecular Biology, Institute of Molecular Genetics and Genetic Engineering, University of BelgradeBelgrade, Serbia; ^3^Department of Biochemistry and Molecular Biology, Faculty of Biology, University of BelgradeBelgrade, Serbia; ^4^Laboratory of Ecology, Systematics, Evolution, UMR 8079, CNRS-UPS-AgroParisTech, University Paris-SudOrsay, France

**Keywords:** resurrection plants, *Ramonda serbica*, *Ramonda nathaliae*, dehydration, rehydration

## Abstract

Paleoendemic species of the monophyletic genus *Ramonda* (*R. myconi*, *R. serbica* and *R.~nathaliae*) are the remnants of the Tertiary tropical and subtropical flora in Europe. They are the rare resurrection plants of Northern Hemisphere temperate zone. *Ramonda serbica* and *R. nathaliae* are chorologically differentiated in the Balkan Peninsula and occupy similar habitats in calcareous, northward slopes in canyons and mountainsides. They remain well-hydrated during spring, late autumn and even in winter. In summer and early autumn when plants are subjected to drought and thermal stress, their desiccation tolerance comes into operation and they fall into anabiosis. Investigations revealed the permanent presence of ubiquitine and its conjugates, high amounts of oxalic acid and proline. Both species are homoiochlorophyllous. It enables them to rapidly resume photosynthesis upon rehydration, but also makes them susceptible to reactive oxygen species formation. Dehydration induces activation of antioxidative enzymes (ascorbate peroxidase, glutathione reductase, polyphenol oxidase), increase in amounts of AsA and GSH, phenolic acids, dehydrins, sucrose, and inorganic ions. Plasma membranes, characterized by high amount of cholesterol, are subjected to decrease in membrane fluidity mostly on account of increased level of lipid saturation. Cytogenetic analysis revealed that *R. nathaliae* is a diploid (2*n* = 48) and probably evolutionary older species, while *R. serbica* is a hexaploid (2*n* = 144). Two species live together in only two localities forming hybrid individuals (2*n* = 96). Polyploidization is the major evolutionary mechanism in the genus *Ramonda* that together with hybridization ability indicates that these relict species which have preserved an ancient survival strategy are not the evolutionary “dead end.”The species of the genus *Ramonda* are promising sources of data important for understanding the complex strategy of resurrection plants’ survival, appraised through a prism of their evolutionary and adaptive potential for multiple environmental stresses.

## INTRODUCTION

The Balkan Peninsula is one of the most important parts within the Mediterranean “hot spot” area and is globally distinguished as a reservoir of biological diversity (Myers et al., 2000). It harbors several desiccation tolerant and preglacial endemo-relict species of the tropical-subtropical family Gesneriaceae. These are the exceptional examples of floristic evolutionary diversification and biogeographical differentiation within South-European eumediterranean and paramediterranean area. Family Gesneriaceae has predominantly pantropical and pansubtropical distribution with only a small number of species extending to temperate regions. Among these are three genera from South Europe. The oligotopic genus *Ramonda* and the monotopic genera *Haberlea* and *Jankaea*, include five species. With the exception of *Ramonda myconi* which inhabits the Iberian Peninsula, all others (*R. serbica* and *R. nathaliae*, *Haberlea rhodopensis,* and *Jankaea heldreichii*) are spread in the Balkan Peninsula. These Tertiary relict and endemic species are perennial, long-lived, and slow-growing poikilohydric plants. They are all evergreen chasmophytic hemicryptophytes and inhabit rock crevices, preferentially in sheltered, rather cool and humid places.

The J.M.C. Richard’s discovery of the Pyrenean *R. myconi* in 1805 was in the latter half of the century followed by the somewhat unexpected finding of *R. serbica* and soon after by that of *R. nathaliae* in the Balkans, by Serbian botanists J. Pančić and S. Petrović. By the end of the 19th century and early in the 20th the detailed descriptions of the Balkan *Ramonda* species, their morphological, ecological and cytogenetic specificities and of their distinguishable habitats, dispelled the initial doubts regarding the taxonomic validity of *R. nathaliae* (Panvčić, 1874; Petrović, 1882, 1885; Košanin, 1921, 1939; Glišić, 1924; Černjavsky, 1928). At the same time they brought to light their unique feature of pokilohydry.

## GEOGRAPHICAL DISTRIBUTION OF BALKAN *Ramonda* SPECIES

**FIGURE 1:**
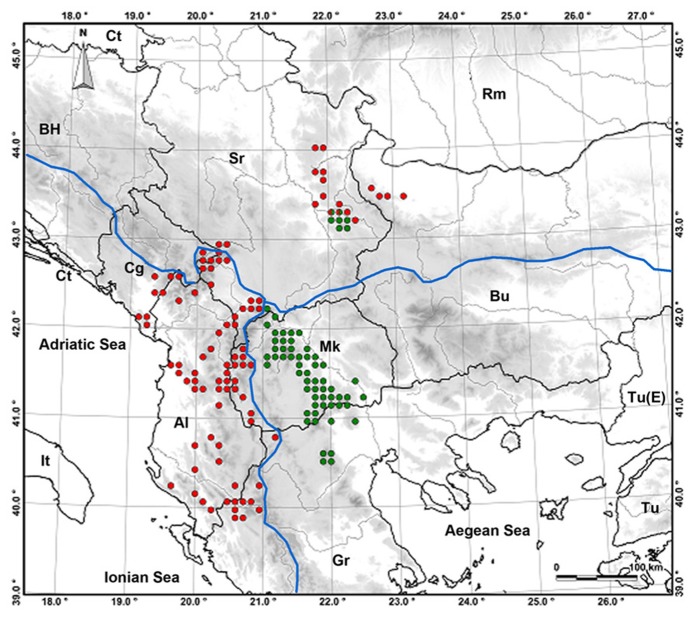
**Distribution map of the genus *Ramonda* in the Balkan Peninsula: *R. serbica* (red dots), *R. nathaliae* (green dots), sympatric populations (red-green dots)**. Blue lines are watersheds between three river systems.

*Ramonda serbica* and *R. nathaliae* occupy different distribution areas and both are almost exclusively calcicole plants (**Figure 1**). *R. serbica* is found in the Southern Balkans and its habitats belong to the Adriatic river system. It spreads in the regions of Albania, Serbia, Montenegro, Macedonia, and Greece, spanning the wide range of altitude from 200 to 1950 m asl. *R. nathaliae* is found in more restricted and rather compact area situated mostly in Macedonia and partially in Greece and in Kosovo. Its altitudinal range is somewhat wider, from 100 to 2200 m asl, and its habitats belong to the Aegean river system (Meyer, 1970; Stevanović et al., 1991). Within this distribution area in only two localities (Pčinja and Raduša gorges) *R. nathaliae* thrives on serpentine soil (Košanin, 1921; Stevanović and Stevanović, 1985).

Both *Ramonda* species have disjunct zones to the north-east (the Central Balkans) of their respective ranges that belong to the Black Sea river system. Within this area, on two localities in South East Serbia (Jelašnica and Sićevo gorges), they form sympatric populations within which they hybridize (Stevanović et al., 1986, 1987; Siljak-Yakovlev et al., 2008a; Lazarević et al., 2013).

## GENERAL ASPECTS OF HABITATS AND ECOLOGY

The sibling species *R. serbica* and *R. nathaliae* are the remnants of the mountain flora that grew in the central and southern Balkans during the late Tertiary, enjoying the subtropical-to-moderate temperature and humidity. The onset of the global cooling of the Northern Hemishere during the Glacial Age reduced their high mountain populations, restricting their settlements to sheltered places such as gorges and ravines in high mountain zone and river gorges and canyons at lower altitudes which offered milder and more stable climatic conditions. In all those places they still thrive as endemic Balkan relicts.

**FIGURE 2:**
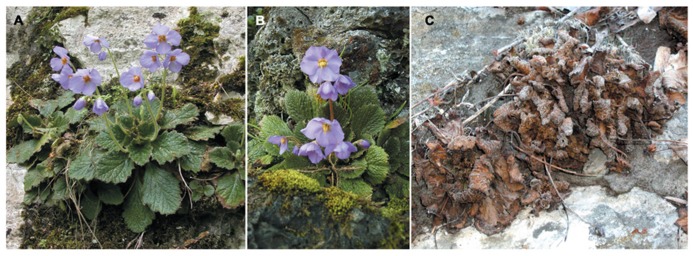
***Ramonda serbica* (A), *R. nathaliae* (B), and *R. nathaliae* individuals in anabiosis (C)**.

Both *Ramonda* species settle in crevices of exclusively north-facing steep rocky sides, sheltered in the shade of northward exposition or of the surrounding forest canopy. The reduced solar energy input in such sites consequently means the lower daily temperature and humidity variations in the habitat which is considered crucial for the maintenance of their activities during the major part of the year. Thus, in respect to similar plants that thrive in tropical/subtropical zone of the Southern Hemisphere, Balkan *Ramonda* species successfully survive in entirely different environmental conditions. These species of the subtropic origin are subjected to continental climate that is characterized by hot and dry summer as well as by winter season with long periods of zero to sub-zero temperatures, often with scanty snow cover. Within this range of climatic extremes, dynamic seasonal weather changes force plants to go through desiccation/rehydration cycles several times during a year (**Figure 2**).

*Ramonda nathaliae* and *R. serbica* grow on the shallow organo-mineral soil covered with dense carpet of mosses. Mosses are very efficient in absorption of water and have high capacity to store it thus helping in improving soil moisture conditions ((Rakić et al., 2009). This type of soil is characterized by conspicuous waterholding capacity based on the hydrophilic character of high amount of organic debris that is also of great significance for maintaning the plant roots in hydrated state. The high economy of *Ramonda* plants is furthermore represented by the efficient recycling of dead outher leaves of the rosette that remain attached to the plant, even though they are already partially decomposed. Thus, the litter in the soil beneath the rosettes is composed mainly of dead *Ramonda* leaves and broken down mosses. The efficacy of the plant water and mineral element uptake is additionaly improved by myccorhyzal fungi that were detected in roots of both *Ramonda* species and whose extraradical hyphae have access to much larger soil volume that often remains inaccessible to the plant roots (Rakić et al., 2009, 2013). Hence, they are of the special importance for plants that grow in crevices on the vertical rocks on thin soil layer and with roots that branch deeply in the bedrock hollows.

Aside from their role in root protection, mosses and litter beneath the plant’s rosette are the high quality cradle for young plants and seedlings. Therefore, the type and the traits of the soil are the prerequisites for the protection of these species survival in extreme habitat conditions.

Clear differences exist between *R. serbica* and *R. nathaliae* with regard to their respective ecoanatomical and ecophysiological characters (Stevanović 1986, 1989–1991; Stevanović and Glišić, 1997; Stevanović et al., 1997/1998). *R. serbica* performs as mesophyte: it grows in fairly humid and warm habitats, situated in rocky outcrops sheltered by surrounding shrubs and small trees, or in the forest understory. In comparison, *R. nathaliae* is a xero-mesomorphic plant: it settles the sites which are exposed to harsher environmental conditions – more warm and dry, and less protected from high irradiance. Its presence on serpentine soil is witness to such pronounced tolerance to adverse effects of climate (extremely high temperature and low humidity) and soil (toxic effects of heavy metal load; Stevanović et al., 1997/1998; Lazarević et al., 2013).

## BIOCHEMISTRY OF DESICCATION TOLERANCE

The critical variable which triggers entering of resurrection plants into anabiosis and their subsequent recovery to full metabolic competence is the availability of water. Slow drying over a longer period, until the complete desiccation sets in, is vital for gradual changes in structure and physiology that require time. Desiccation in *Ramonda* plants from well hydrated to completely dehydrated state takes place in about 15 days and quite noticeably falls into two successive, but different stages. The initial desiccation period is longer (7–10 days) when plant relative water content (RWC) diminishes to about 40–50%. This is followed by the short period of drastic water loss and finally results in complete plant desiccation (RWC < 10%). The prominent increase in some physiological parameters, such as increase in activity of enzymes involved in antioxidative protection, at RWC of about 30–70% indicate that *Ramonda* plants suffer from the strong, but transitory metabolic disturbances that occur in moderatelly hydrated plants, during both dehydration and rehydration (Sgherri et al., 2004; Jovanović et al., 2011). This can be correlated with the redox shifts induced by metabolic imbalances that are the source of signals which lead to the coordinated activation or cessation of the defense mechanisms necessary for the survival of desiccation (Jovanović et al., 2011).

At the beginning of rehydration, the recovering plant goes through an oscillatory, unstable and vulnerable short period. Generally, in about a day’s time the markers of the oxidative stress subside, and the plant regains satisfactory stability in about 48 h, when the RWC reaches control values. However the fine-tuning synthesis and repair could be seen 6 days away from the beginning of rehydration.

### ANTIOXIDATIVE RESPONSE

**FIGURE 3:**
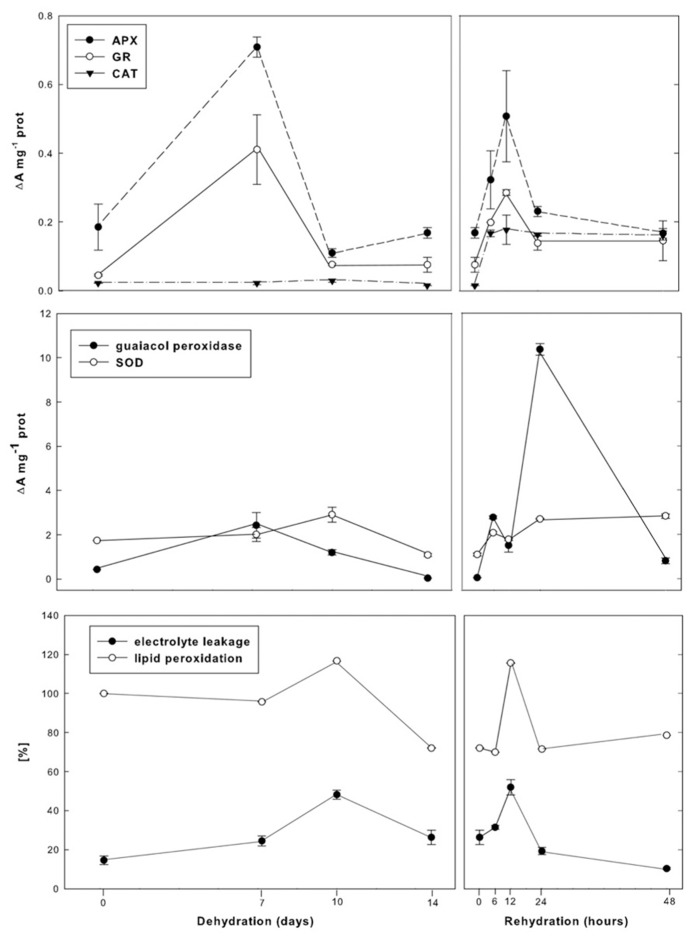
**Changes in APX, GR, CAT, SOD, and guaiacol peroxidase activities as well as peroxidation level and electrolite leakage during dehydration and rehydration of *R.nathaliae***.

The general adaptive strategy for surviving numerous repetitive dehydration/rehydration cycles during the life of resurrection *Ramonda* plants is based to a large degree on their ability to limit cellular damages during desiccation and rewatering. In parallel with changes in metabolic activities, *R. serbica* and *R. nathaliae* induce high antioxidant activity that protect cells from elevated production of reactive oxygen species (ROS), which is one of the most deleterious consequences of water deficit and metabolic disbalances. This is especially pronounced in early dehydration as well as after several hours of rewatering (30–70% RWC). These phases, when plants are moderately hydrated, are characterized by very high activities in superoxide dismutase (SOD), ascorbate peroxidase (APX), and glutathione reductase (GR) which indicates redox potential shifts in cells induced by increased generation of ROS (Sgherri et al., 2004; Jovanović et al., 2011; **Figure 3**).

Ascorbate peroxidase and GR are the first enzymes with markedly increased activities during initial dehydration and with similar profiles, suggesting that ascorbate-glutathione cycle plays a major role in maintaining redox homeostasis. At the same time, activities of non-specific peroxidases (PODs) are also elevated playing a role not only in ROS detoxification, but also in cell wall remodeling. Further dehydration leads to decrease in APX, POD, and GR activities, but also to transient increase in SOD activity that consequently results in enhanced generation of H_2_O_2_ and transient increase in membrane lipid peroxidation and ion leakage through plasmalemma. Lastly, in severely dehydrated plants and in the state of anabiosis, when metabolism is almost completely suspended, production of H_2_O_2_ is reduced and the antioxidant activities of all investigated enzymes are close to their control levels. This indicates their resistance to breakdown, even under conditions of nearly complete water loss.

Increased H_2_O_2_ formation that occurs during the later phases of dehydration is the signal for the multiple increments in ascorbate and glutathione pools (Augusti et al., 2001; Sgherri et al., 2004). In desiccated plants both pools are composed of their reduced forms, AsA and GSH, whose role is to maintain the redox homeostasis during the latest phases of dehydration, in desiccated plants and especially in rehydration. The most of this large AsA and GSH reserve is used almost immediately upon rewatering, thus metabolizing H_2_O_2_ through ascorbate-glutathione cycle. As a consequence, during the first several hours of rewatering, their pools, now comprised mainly of their oxidized forms, drastically decrease, so that the transient increase in lipid peroxidation level and electrolyte leakage through plasmalemma occur. At that time, the coordinated high activities of SOD, CAT, APX, GR, and PODs are present, indicating that the first few hours of rehydration represent extremely dramatic period regarding cellular oxidative stress (Sgherri et al., 2004; Jovanović et al., 2011).

Another group of compounds with pronounced antioxidant activity found in unusually elevated amount in leaves of *R. serbica* are phenolic acids (Sgherri et al., 2004). The most representative phenolic acids are chlorogenic acid, protocatechuic acid, and *p*-hydroxybenzoic acid. The very high activity of phenolic peroxidases and non-specific POD as well as decrease in total amount of phenolic acids in the course of dehydration indicate the strong oxidation of phenolic acids and suggests that they act against ROS, functioning as substrates for peroxidases (Sgherri et al., 2004; Veljović-Jovanović et al., 2008). In addition, high activity of polyphenol oxidase (PPO) in desiccated leaves of *R. serbica* show that oxidation of phenolics plays an important role in the adaptation mechanism to water deficit (Veljović-Jovanović et al., 2008). On the contrary, during the first few hours of rehydration, the specific phenolic POD activity is low and the phenolic acid contents increase, the most probably due to the strong oxidation of the abundant ascorbate pool. Rehydration brought about short-term disappearance of the PPO isoforms (with pI from 5 to 7.4) that are normally present in desiccated leaves, which were than re-induced within 1 day upon rehydration. This observation is in accordance with the transient decrease of SOD, APX, and POD activities that occur during the first few hours of rehydration (Veljović-Jovanović et al., 2006). The transient inactivation of the antioxidative enzymes can be the consequence of uncontrolled radical chain reactions, provoked by small increase in RWC, leading to oxidation of the proteins (Davies et al., 1987).

### PHOTOSYSTEM II PHOTOCHEMICAL EFFICIENCY

Both *Ramonda* species retain about 50% of chlorophyll in desiccated state and therefore are typical homoiochlorophyllous desiccation-tolerant (HDT) plants (Dražić et al., 1999; Jovanović et al., 2011). In the course of dehydration leaves progressively fold inward so that the pronouncedly pubescent abaxial leaf side becomes exposed to the sunlight. Thus, the chloroplasts in the palisade tissue remain shaded and protected from the light, minimizing the possibility for the light induced damage of photosynthetic apparatus in water deprived cells. In this manner, morphological changes observed in desiccated plants represents indispensable mechanism for protection from photooxidative damages. The importance of leaf folding was shown in homoiochlorophyllous resurrection plant *Craterostigma wilmsii.* When its rosette leaves were prevented from folding during dehydration in light, the lethal damages induced by light stress occurred and resulted in the loss of the plant viability (Farrant et al., 2003).

At the beginning of dehydration of *R. serbica* the photochemical efficiency of PS II decreases slowly, but than drops sharply at RWC values below 40%, as found also in *Haberlea rhodopensis* (Augusti et al., 2001; Peeva and Cornic, 2009). The reduced rates of chlorophyll *a* fluorescence and photosynthetic electron transport in dehydrating plants consequently lead to increase in excessive excitation energy that is finally dissipated as heat, thus preventing from photoinhibition (Eskling et al., 1997; Niyogi, 1999; Smirnoff, 2000). The observed transient increase in non-photochemical quenching (NPQ) during the mild water deficit is enabled by accumulation of carotenoid zeaxanthin (Augusti et al., 2001). The de-epoxidation state of xanthophyll cycle (DPS) increases during dehydration and exhibits the similar pattern of variation as NPQ. Thus, in dehydrated plants zeaxanthin and antheraxanthin representes from 60 to 80% of the violaxanthin + antheraxanthin + zeaxanthin pool (Augusti et al., 2001). Afterward, at RWC below 30%, when leaves are mostly folded, the NPQ declines.

Zeaxanthin that is retained in dehydrated leaves plays a role in non-radiative energy dissipation at the beginning of rewatering when PS II centers are still in recovery. At that time, at low RWC and low electron transport rates, the excitation energy is still in excess and therefore it is dissipated as heat, which is confirmed by high DPS and NPQ (Augusti et al., 2001). The almost completely recovered photochemical efficiency of PS II within the first 35 h of rewatering suggests that thylakoid membranes mostly restored their structure and functionality.

### MAINTENANCE OF THE CELL INTEGRITY

#### Lipids

The total polar lipid content in leaves of both *Ramonda* species is rather low (15–20 mg/gDW) compared to what is usually found in other flowering plants (Stevanović et al., 1992). In well hydrated plants the galactolipid monogalactosyl-diacylglycerol (MGDG) is the main lipid class in both *Ramonda* species, followed by digalactosyl-diacylglycerol (DGDG). Desiccation reduces galactolipid content to about 10% of its control value and induces changes in relative proportions of MGDG to DGDG, being in desiccated plants in favor of DGDG. The later have positive effect on maintenance of the chloroplast membranes in a bilayer conformation that is necessary for protection of its functions (Dörmann and Benning, 2002).

The striking feature of plasma membrane lipids is the abundance of free sterols (FSs) that account for more than half of plasmalemma total lipid content, irrespectively of the plant water content (Quartacci et al., 2002). Severe water depletion causes reduction in almost 75% in the plasma membrane lipid content which is based mainly on decrease in phospholipids amount. This consequently leads to the reduction in plasma membrane area, thus accompanying the desiccation-induced shrinkage of the cell volume. Regardless of the RWC, the major plasmalemma FS is cholesterol. Its already high amount in well watered plants doubles in the course of dehydration, reaching even 28 mol% in the PM FS amount in completely desiccated leaves (Quartacci et al., 2002). In dehydrated cells cholesterol that interacts with membrane phospholipids functions as “glue” and stabilizes the membrane and proteins within the membrane (Yoshida and Uemura, 1990; Quartacci et al., 2001). The additional positive effect on membrane stability is achieved by increase in level of cerebrozides (CER) and decrease in the unsaturation level of individual phospholipids and total lipids. This improves stability between lipids and membrane-intrinsic proteins and increases the membrane transition temperature (Quartacci et al., 2002). The reduced membrane fluidity in plants with low water contents reflects in very low values of injury index, obtained from electrolyte leakage through plasmalemma, and low lipid peroxidation in both *Ramonda* species (Jovanović et al., 2011). All detected variations in the lipid content prevent extremes in the consistency of the cell membrane, stabilize its structure and preserve its biological functions during dehydration and rehydration as well as during the annual temperature variations.

#### Sugars

The dominant soluble carbohydrate in *R. serbica* leaves is sucrose (Živković et al., 2005). Its amount markedly increases in the course of dehydration, when it might perform several different roles in cells: (1) in osmoregulation, when together with proline and inorganic ions (K, Ca, Mg, and Na) participates in water retention in cells (Živković et al., 2005); (2) in conservation of the cell membranes in desiccated state (Crowe and Crowe, 1986; Crowe et al., 1987; Williams and Leopold, 1989; Allison et al., 1999); (3) as the source of energy in the initial phase of rehydration, untill the full photosynthetic acitivity restores.

#### Amino acids

The most abundant amino acid detected in leaves of *R. serbica* is proline and in different phases of dehydration and rewatering it represents from about 50% to even 70% of the total free amino acid pool (Živković et al., 2005). During water deficit the amounts of total free amino acids and proline markedly decreased. This is, the most probably, the consequence of changed balance between their biosynthesis and catabolism. In further dehydration, at RWC lower than 20% proline markedly accumulates, but upon rewatering its amount suddenly decreases. The pattern of changes in proline content during dehydration and rehydration is similar to those detected in ascorbate and glutathione pools (Jovanović et al., 2011) pointing to its possible role in antioxidative defense in the initial phase of rehydration, but also to its role in osmoregulation, stabilization of subcellular structures and in gene expression (Vanrensburg et al., 1993; Iyer and Caplan, 1998; Hare et al., 1999; Hong et al., 2000; Kavi Kishor et al., 2005).

#### Organic acids

The major organic acid in both leaves and roots of *R. serbica* is oxalic acid. It corresponds to even 90% of all detected organic acids along dehydration–rehydration cycle (Živković et al., 2005). It can be suggested that degradation of the large ascorbate pool upon plant rewatering is the main source of oxalic acid. In *Ramonda* plants that accumulate K, Na, Ca, and Mg ions along desiccation it might play a role in the maintenance of ionic balance.

#### Ubiquitin and dehydrins

Pronounced water deprivation in cells has negative effects on proteins conformation and their activities, but also leads to the synthesis of new polypetides involved in the protection of cell structures. The ubiquitin is considered a stress protein that acts as a tag for selective degradation of short-lived, denaturated, incomplete or misfolded proteins via 26S proteasome (Kurepa and Smalle, 2008). Although the desiccation-related effects on level of ubiquitin and its conjugates are poorly understood, our results demonstrate that level of protein ubiquitination is increased during dehydration phase D_2_ (RWC 55%) and might be the result of higher proteolysis rate by the 26S proteasome caused by more pronounced water depletion (Jovanović et al., 2010). The observed increase of ubiquitin-tagged proteins indicates the higher level of protein turnover, suggesting that midle phase of dehydration (D_2_) can be critical for induction of desiccation adaptive response. Relatively unchanged level of protein ubiquitination during rehydration of *R. nathaliae* suggests the predominance of desiccation specific protein preservation mechanisms over its degradation and *de novo* synthesis. The important role in protein protection could be attributed at least in part to dehydrins. The western blot analysis in *Sporobolus stapfianus* showed that maximum in rehydration-associated transcripts accumulation coincided with depletion of ubiquitin monomer, which directly indicates an increase in protein degradation (O’Mahony and Oliver, 1999). However, changes in the level of ubiquitin conjugated proteins can be a reflection of altered rates of ubiquitination, deubiquitination, or proteolysis by the 26S proteasome, leading us to further analysis, including analysis of ubiquitin transcript, which are required before some definite explanations of observed changes are made.

Another mechanism meritorius for desiccation-tolerance is represented by dehydrins – the subgroup of LEA proteins. A positive correlation between accumulation of dehydrins and adaptation to cellular dehydration has been extensively documented in the literature. In our study, we found that considerable amounts of dehydrins were present already in fully hydrated leaves and that some of them were upregulated during dehydration/rehydration cycle (Jovanović et al., 2011). Considerable amounts of dehydrins in control plants may indicate that they are all required for normal plant metabolism, although their higher amounts are required during dehydration. Thus, in the drought-tolerant resurrection plant *Craterostigma plantagineum* two proteins that resemble dehydrins were detected in well-watered leaves, while changes in plant hydric status led to appearance of new proteins (Schneider et al., 1993). In adition, dehydrins found in *Ramonda* plants could be responsible for water retention enabling slow dehydration that is necessary for activation of various protective mechanisms. Our result might stand for hypothesis that some of resurrection plants have a pool of previously sinthesized proteins, which serve as protectants. Emergence of the new dehydrins during dehydration coincides with both decline in antioxidative enzymes activities and lipid peroxidation, suggesting their role in protection of cell structures under conditions of significant water loss. Also, this indicates their possible free radical scavenging activity as it has been previously reported for *Citrus unshiu* dehydrin CuCOR19 (Hara et al., 2004). Different pattern of dehydrins during rehydration indicated their new roles – possible chaperone-like activity in refolding and repairing of proteins. The observed changes in expression of dehydrins suggest the need for coordinated and tightly regulated expression of individual dehydrins with specific function during dehydration/rehydration. In addition, dehydrin expression has to be coordinated with activities of other participants in *R. nathaliae* desiccation tolerance, such as antioxidative enzymes. Our results showed that down-regulation of antioxidative enzymes is associated with up-regulation of dehydrins.

## POLYPLOIDY AND HYBRIDIZATION AS THE MAIN MECHANISMS IN THE EVOLUTION OF THE GENUS *Ramonda*

Special attention in recent investigations is focused on cytogenetical and genome size studies of all three species of the genus *Ramonda*, because they promise to bring new insight into inter- and intra-specific variations, into the genetic relationship between these species and the outcome of their hybrids.

According to our results *R. myconi* and *R. nathaliae* are diploids with 2*n* = 2*x* = 48 (Siljak-Yakovlev et al., 2008a) and this is in agreement with previous literature data (Ratter and Prentice, 1964; Contandriopoulos, 1966; Lepper, 1970). These two species also have the similar genome size, *R. myconi* 2C = 2.59 pg and *R. nathaliae* 2C = 2.32 pg (Siljak-Yakovlev et al., 2008a). The most complex of three species is *R. serbica* for which different results about the chromosome number can be found in the literature, from 2*n* = 72 (Glišić, 1924; Daskalova et al., 2012), 2*n* = 96 (Contandriopoulos, 1966; Daskalova et al., 2012) to 2*n* = 108 (Daskalova et al., 2012). In our investigations of genome size and/or chromosome number in 18 populations of this species from different parts of its distribution area mostly hexaploids with 2*n* = 6*x* = 144 chromosomes and with average genome size of 2C = 7.91 pg are found (Siljak-Yakovlev et al., 2008a; Lazarević, 2012; Lazarević et al., 2013). Few octoploid (2*n* = 8*x* = 192) and decaploid individuals (2*n* = 10*x* = ~230) are discovered in only one population from Montenegro (Siljak-Yakovlev et al., 2008a).

Interestingly, two diploids have different monoploid genome sizes (1C*x*). *R. nathaliae* has lower value of monoploid genome size (1C*x* = 1.16 pg) than diploid *R*. *myconi* (1C*x* = 1.30 pg) and hexaploid *R. serbica* (1C*x* = 1.32 pg), which have very similar values.

Cytogenetically the most complex *Ramonda* populations are the two only existing sympatric populations where *R. nathaliae* and *R. serbica* grow together, both in SE Serbia at the localities Radovanski Kamen and Oblik. Extensive genome size and chromosome number analyses in these populations revealed the existence of hybrid individuals. Most of these plants are tetraploids with 2*n* = 4*x* = 96 and average genome size of 2C = 5.14 pg (Siljak-Yakovlev et al., 2008a; Lazarević, 2012). Thus, hybrid individuals are characterized by intermediary chromosome number and intermediary genome size compared to parent species. However, several cases of individuals with larger genome sizes have been found in sympatric populations. Such individuals could originate from backcross of hybrids with *R. serbica* (2C~6 pg), while potential octoploids (2C~9.5 pg) could result from the spontaneous genome duplication of tetraploids or from joining of unreduced gametes of *R. nathaliae* and *R. serbica* (Siljak-Yakovlev et al., 2008a).

**FIGURE 4:**
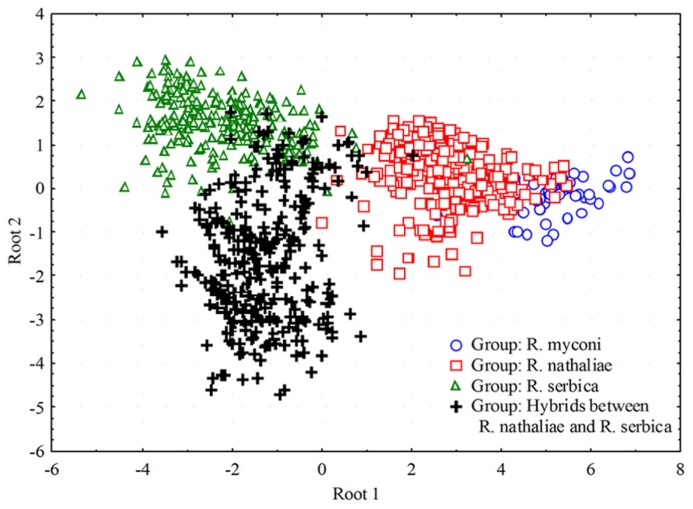
**Results of Canonical Discriminant Analysis (CDA) of pollen characters plotted along the first two discriminant axes for three *Ramonda* species and hybrids between *R.nathaliae* and *R. serbica***.

Hybrids are also confirmed by the detailed pollen and seed analyses (Lazarević et al., 2013). While pollen grains of *R. nathaliae* and *R. serbica* are always 3-colporate and uniform in size, pollen from hybrids is very heterogeneous both in number of colpi (3-, 9- and 12-colporate) and the size of the grains (**Figure 4**). Seeds from hybrid individuals are very small, 2–3 times smaller than those from parental species and germinate weekly (c. 1%).

Because tetraploid hybrid individuals inherit one part of the genetic informations from the diploid *R. nathaliae* and three parts from hexaploid *R. serbica*, it is expected that hybrids morphologically look more like *R. serbica*. Hence, in the field it is very difficult to distinguish hybrids from parents, in particular from *R. serbica*. Detailed morphological analyses of leaves and flowers from both species and from the hybrid individuals, now in progress, might reveal morphological parameters discriminative for hybrids and could possibly point to inheritance mechanisms of certain morphological characters (Lazarević et al., 2012).

Although *R. nathaliae* and *R. serbica* are considered as relict species, several factors contribute to hybridization between them: spatial proximity of individuals from two species in sympatric populations, similar flowers with the same flowering time, the same pollinators. Thus, the existence of hybrid individuals between *R. nathaliae* and *R. serbica* suggest that completely effective barriers for their intercrossing are still not in place (Lazarević et al., 2013) and reveals complex processes of hybridization, introgression, and genome duplications (Siljak-Yakovlev et al., 2008b).

**FIGURE 5:**
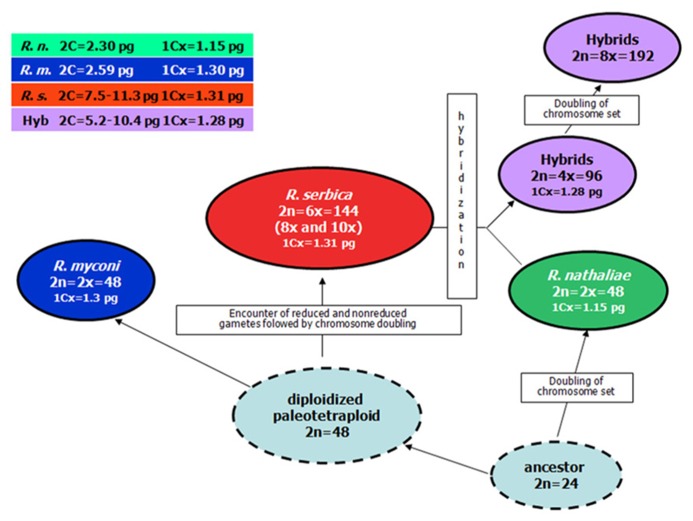
**Hypothetical scheme of the evolution of polyploidy in the genus *Ramonda***.

Based on cytogenetical results, polyploidization is the major evolutionary mechanism in the genus *Ramonda*. Because the basic chromosome number in *Ramonda* is quite high (*x* = 24), it is probable that the two diploids *R. myconi* and *R. nathaliae* are paleopolyploids. Thus, based on chromosome numbers and monoploid genome size, a common ancestor with 2*n* = 24 possibly gave firstly *R. nathaliae* and than paleotetraploid from which after diploidization *R. myconi* and *R. serbica* evolved (**Figure 5**; Siljak-Yakovlev et al., 2008a).

The most recent results from molecular AFLP analysis confirmed clear taxonomic differentiation of *R. nathaliae* and *R. serbica* (Lazarević, 2012). Low genetic diversity is expected, because this is characteristic of many species with the limited distribution (Hamrick and Godt, 1996; Ge et al., 1999). In *R. myconi* from Iberian Peninsula relatively high levels of genetic diversity were detected by RAPD analyses especially within populations, suggesting isolation by distance and existence of “refugia within refugia” (Dubreuil et al., 2008).

## CONCLUSION

Considerable amount of knowledge exists about flowering desiccation tolerant plants that thrive in tropical/subtropical zone of the Southern hemisphere, which is considered their natural “home.” In respect to them, *Ramonda* species successfully survive in entirely different environmental conditions, coping not only with high temperatures and water deficit, but also with low winter temperatures, and in some cases, with heavy metal overload.

According to data obtained in screening of different protective processes that are summarized in this article, we could withdraw the key mechanisms in desiccation tolerance of *R. serbica* and *R. nathaliae*. The main roles in these processes appertain to dehydrins and dehydrin-like proteins, non-reducing sugars, ROS and mechanisms of redox control. Considerable amount of dehydrins that are already present in fully hydrated leaves points to their possible role in water sequestration, thus enabling slow dehydration and consequently alleviating stress. The water loss induces severe metabolic imbalances and enhanced production of ROS such as H_2_O_2_. The ROS could activate redox sensitive mechanisms that lead to transcriptional reprogramming.

Further investigations should be focused on conservation processes – cell ultrastructural changes, physico-chemical mechanisms underlying protection of cellular components, and genes which orchestrate all activities during dehydration/rehydration.

Today, the ancient paleopolyploid species *R. nathaliae* is the only European representative of Gesneriaceae that successfully persists on serpentine soil characterized by high heavy metal content, high summer temperatures and low humidity as well. In these conditions, their plants have smaller rosettes, smallest pollen grains with thinnest exine and lower viability, smallest seeds and even smallest genome size in comparison with plants of these species growing on limestone substrate (Lazarević et al., 2013). The highly effective protective mechanisms, primarily involved in desiccation tolerance, most likely enable it to tolerate heavy metal overload and disbalances in nutrients (Rakić et al., 2013). These fundamental mechanisms apparently enable them the cross-tolerance of various environmental stress conditions, such as low winter temperatures. It would be interesting to compare responses of diploid *R. nathaliae* and hexaploid *R. serbica* to different kinds of abiotic stress because the increased tolerance of polyploids to environmental stress has been frequently reported (Zhang et al., 2010).

Because of the lower genetic diversity, longevity of adults and vulnerability of seedlings, *Ramonda* species can be more sensitive to the environmental stress of the recent climatic changes. Therefore, they must be adequately protected, primarily through the protection of their habitats (Picó and Riba, 2002; Lazarević, 2012). Special attention should be devoted to the sympatric populations where the evolutionary process, concerning two relic Balkan species *R. nathaliae* and *R. serbica* living together and forming hybrid individuals, is still ongoing. All this indicates that these “living fossils” which have preserved an ancient survival strategy are not the evolutionary “dead end.” On the opposite, they represent outstanding organisms for scientific investigations and revelation of the secret of successful survival of desiccation.

## Conflict of Interest Statement

The authors declare that the research was conducted in the absence of any commercial or financial relationships that could be construed as a potential conflict of interest.
